# Supplementary material for: Quantitative Spatiotemporal Mapping of Lipid and Protein Oxidation in Mayonnaise
Source: Antioxidants (Basel). 2020 Dec 15;9(12):1278. doi: 10.3390/antiox9121278 (PMC7765159; doi:10.3390/antiox9121278)
Supplement: Supplementary file 1 [file antioxidants-09-01278-s001.pdf]

Quantitative spatiotemporal mapping of lipid and protein oxidation in mayonnaise

Suyeon Yang[1], Aletta A. Verhoeff[2], Donny W.H. Merkx[1, 2, 3], John P.M. van Duynhoven[1, 2, *] and Johannes Hohlbein[1, 4, *]

Supplementary material

Figure S1. (**A**) CLSM images of four different mayonnaises (sample I-IV) in different random positions. All samples showed the same tendency as Figure 1B. (**B**) The same experiment was done in an independent carrier. The image acquisition conditions are slightly different compared to the main figure (Fig.1). Scanning format was 1024x1024 pixels (62 μm by 62 μm) and the line-scanning speed was set to 100 Hz. The sample still shows the same tendencies in lipid and protein oxidation. Images were combined from three different channels taken with λ_{ex}640 nm/λ_{em}660-750 nm (red), λ_{ex}561 nm/λ_{em}580-660 nm (green) and λ_{ex}488 nm/λ_{em}500-560 nm (blue).

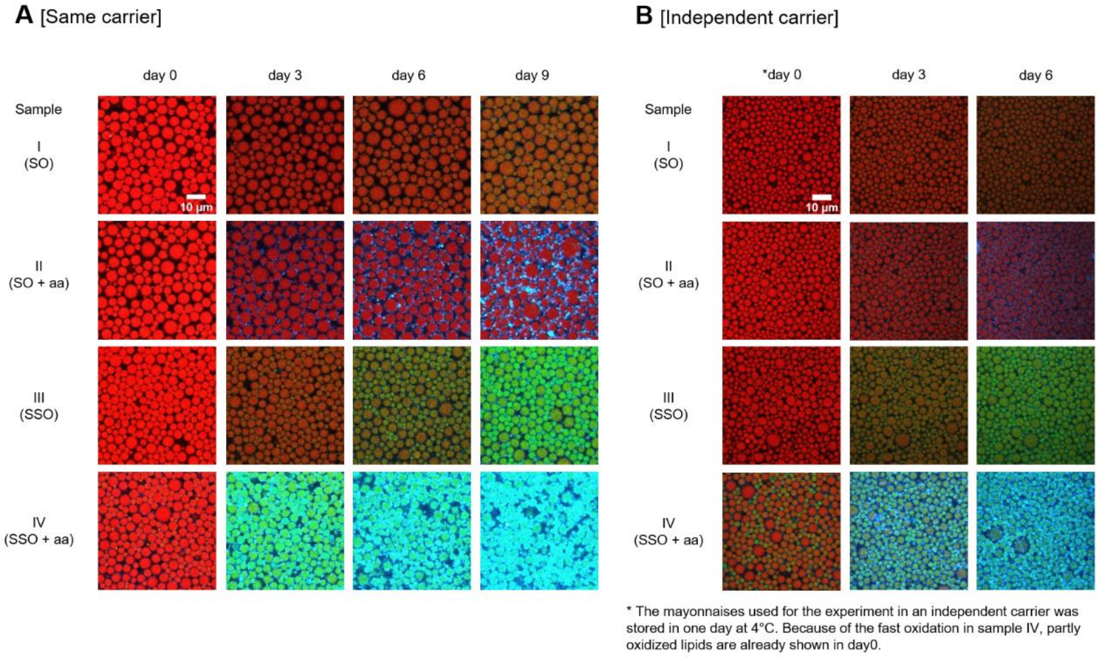

Figure S2. The spectral changes of BODIPY665/676 in native oil and after retrieving the oil from the mayonnaise. Fluorescence emission spectra were measured for excitation at 488 nm, 561nm 640nm (colored vertical dashed lines). Colored areas show the range of detection. Blue channel: 500 nm – 560 nm, green channel: 580 nm – 660 nm, and red channel: 660 nm – 750 nm. (**A**) Native soybean oil (SO, black line) and native stripped soybean oil (SSO, magenta line) after 0 day (straight lines) and 15 days (dashed lines). In both native soybean oil (SO) and stripped soybean oil (SSO), the initial FL intensity at day 0 was similar in all channels with a dominant emission peak around 680 nm after excitation at 640 nm. A slightly smaller intensity of SSO than SO in the blue channel reflects the depletion of tocopherol by stripping the oil. After storing the samples at 30℃ for 15 days, only SSO showed a small increase of emission in the green channel, indicating the presence of oxidation. (**B**) Retrieved oil from freeze-thawn emulsions after 0 days (straight line). Soybean oil (SO, black line), Soybean oil with added ascorbic acid (SO + aa, blue line), and stripped soybean oil (SSO, magenta line). They showed higher FL intensities in the green channel than the bulk oil samples due to the presence of pro-oxidants in emulsifiers. On day 0, SO and SO + aa showed slightly higher intensities than SSO in the blue channel because of the removed molecules as we already observed in S. Fig. 1a. In the green channel, FL intensity of SSO was higher than SO suggesting that SSO was more prone to oxidation and SO + aa had similar intensities as the SO sample. In the red channel which shows the non-oxidized lipids, SO and SO + aa had a higher intensity than SSO. (**C**) Retrieved oil from freeze-thawn emulsions after 13days (dashed line). After 13 days, the SSO sample showed a higher degree of oxidation than SO reflected by a higher intensity in the green channel and a larger drop of emission in the red detection channel. We further observed an unexpected peak around 530 nm in the blue channel in all samples, which we attribute to a second state of the oxidized BODIPY fluorophore. SO + aa showed a lower response with the green intensity of SO being 6 times higher than SO + aa thereby indicating that ascorbic acid has a role as an anti-oxidant on lipids. In stripped soybean oil, however, emulsions with ascorbic acid had a higher oxidation rate than without. This is because of the combined effects of tocopherol removal by stripping the oil and the presence of ascorbic acid in the aqueous phase.

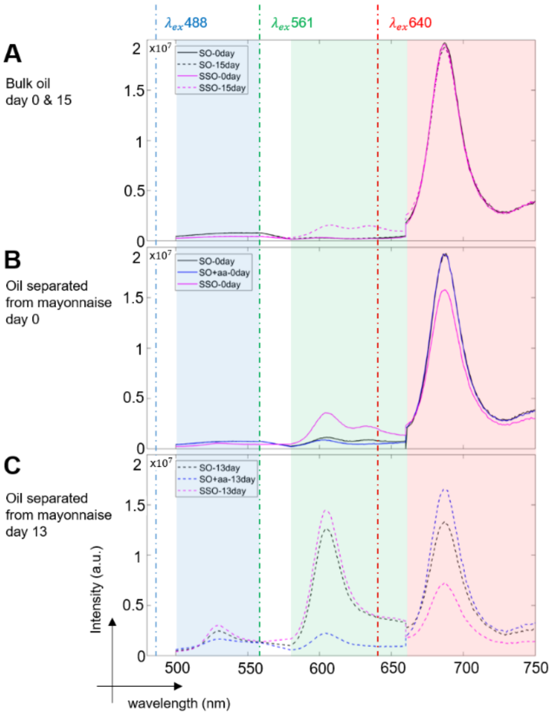

Figure S3. Original data and images after segmentation from Figure 1D. R: red channel, ex 640nm, G: green channel, ex 561nm, B: blue channel, ex 488nm. To show the lipid and protein oxidation, we combined green and blue channels.

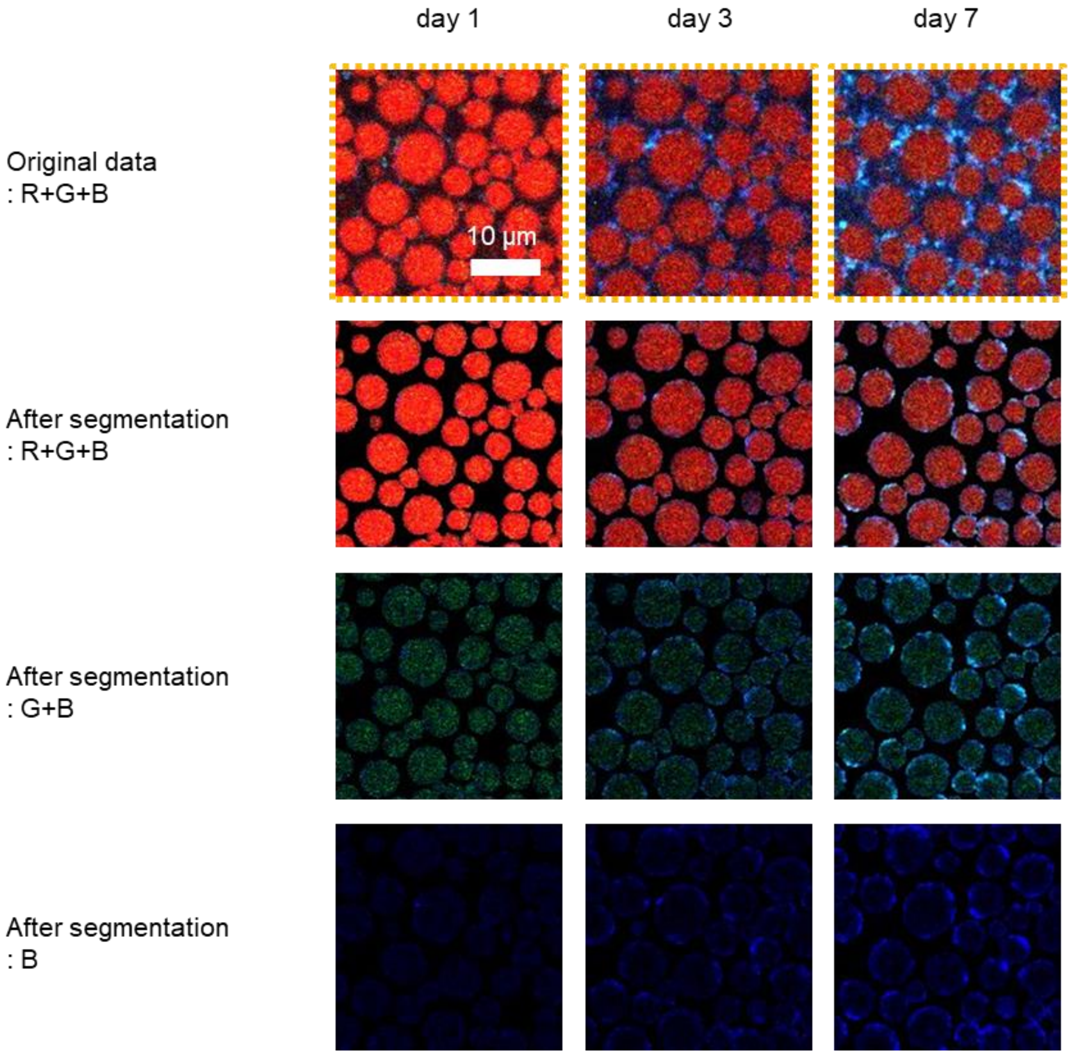

Figure S4. Re-assigning process after using Trackmate. As only a single blob diameter can be chosen for tracking analysis in Trackmate, one tracked number can be assigned in several droplets. (**A**) Example of overlapping tracking numbers. In this case, the droplet which has a larger area in the tracking number was chosen. (**B**) Tracked numbers from other droplets (Trackmate) can be assigned in non-tracked droplets (Stardist). One of them is randomly chosen if it is assigned with the same tracking area. Those are removed in the final steps by choosing the only droplets which are fully tracked for 10 days. (**C**) The final number of droplets for sample I-III after Trackmate and re-assigning process.

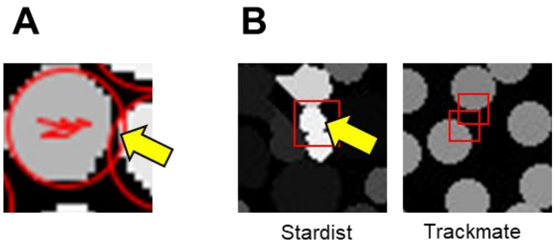

C

	Final numbers of droplets		
Sample	I	II	III
Trackmate	589	597	859
Re-assigned	484	445	641

Figure S5. Statistical analysis of fluorescence intensity changes from confocal images. Fluorescence changes of oxidized and non-oxidized lipids in oil droplets. (**A**) For oxidized lipids in oil droplets, sample I (SO) showed a slow oxidation rate (2.43 photons per pixel on day1, 5.68 on day10) but was oxidized faster than sample II (SO + aa) with 3.15 on day 1 and 3.94 on day 10. Sample III (SSO) had highly increased FL after 6 days (day1: 3.72, day 6: 6.82, and day 10: 13.97) and protein oxidation started to increase as well. On the other hand, sample II (SO + aa) had a high level of protein oxidation but it didn't influence lipid oxidation. (**B**) For non-oxidized lipids in oil droplets, FL intensity of sample I and II (SO and SO + aa) is decreased from 20.86 to 8.97 and from 19.05 to 8.42 during 10days, respectively, and sample III (SSO) showed a higher decrease rate from 18.98 to 2.43 photons per pixel.

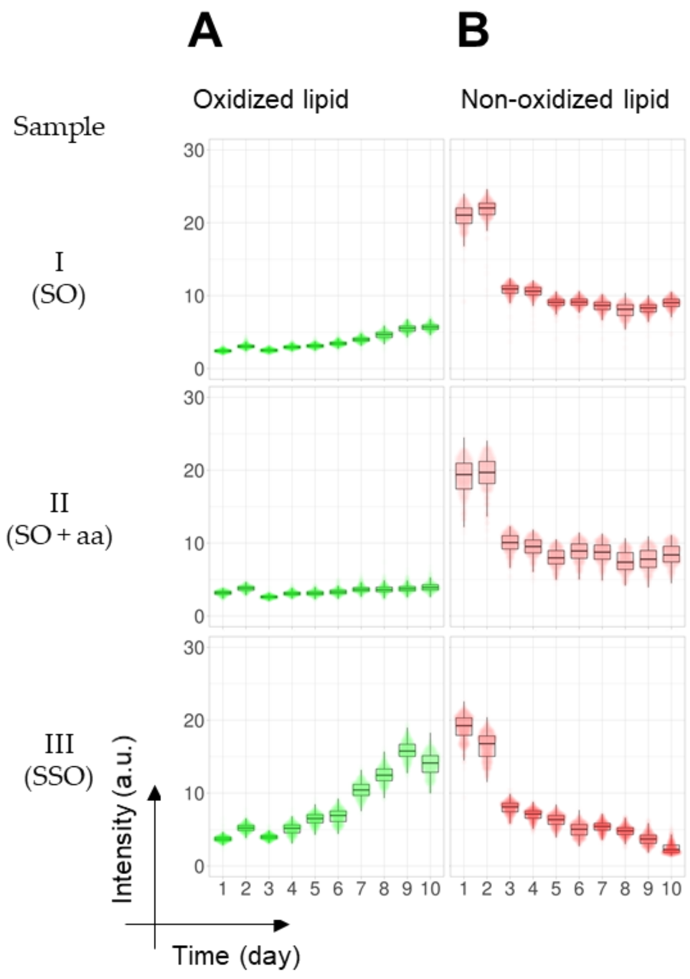

Figure S6. Gompertz fitting on Figure 3A and Figure S3A. The initial value of the asymptote in the Gompertz curve, a, was set to 100 in the main figure and the fitted curves are shown in Figure S6A. The fitted curves and growth rate with the initial fitting value of 20 for protein oxidation and 30 for lipid oxidation are shown in Figure S6B, C. The tendency of growth rate depending on the radius is similar between two different initial values. The final fluorescence intensity at the endpoint of oxidation corresponding to the asymptote, a, is difficult to set because of the structure deformation in mayonnaise upon oxidation. Instead, we fitted with two different initial values.

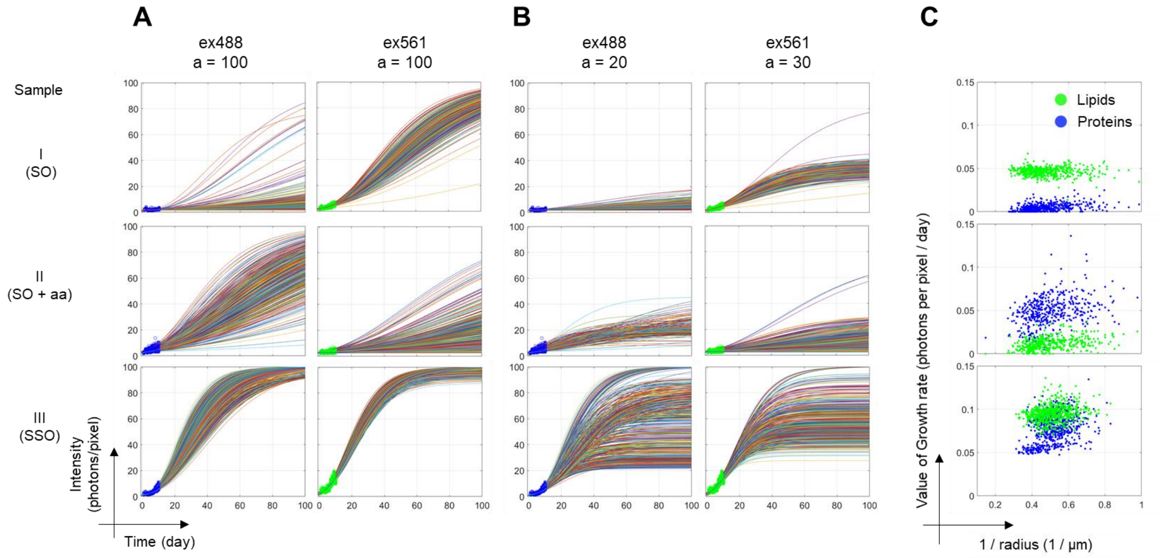

Figure S7. Diffusion measurements of BODIPY 665/676. Dye diffusion was tested by mixing dyed and undyed mayonnaise with the ratio 1:1. **(A)** CLSM images and their histograms (red (non-oxidized) channel only) of mixtures of dyed and undyed soybean oil mayonnaise (SO). The image size is 80 μm x 80 μm. Samples were stored at 22℃. **(B)** Peak positions of image histograms of mixtures of dyed and undyed mayonnaise as a function of time.

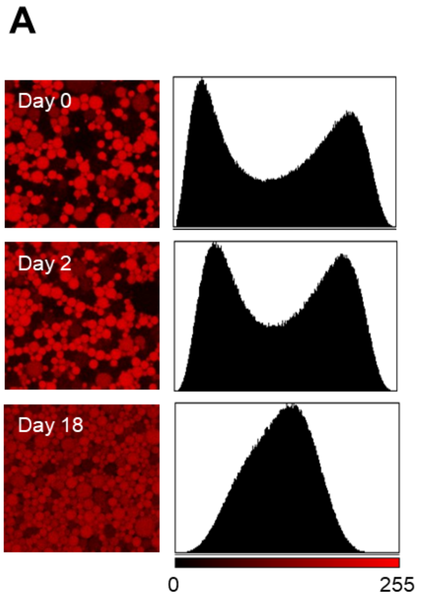

Table S1. Statistical analyses result of ANCOVA (analysis of covariance) on ***protein*** oxidation rate with the antioxidant effect and the inverse of the droplet radius.

Analysis of Variance

Source	DF	Sum of Squares	Mean Square	F Ratio
Mode	5	0.9309	0.1862	4805.899
Error	1564	0.0606	0.000039	Prob > F
C. Total	1569	0.9915		**<.0001***

Effect Tests

Source	Nparm	DF	Sum of Squares	F Ratio	Prob > F
Sample	2	2	0.8597	11095.37	<.0001*
1/radius	1	1	0.0076	195.4081	<.0001*
1/radius * sample	2	2	0.0036	46.3865	<.0001*

Table S2. Statistical analyses result of ANCOVA (analysis of covariance) on ***lipid*** oxidation rate with the antioxidant effect and the inverse of the droplet radius.

Analysis of Variance

Source	DF	Sum of Squares	Mean Square	F Ratio
Mode	5	1.1367	0.2273	13818.83
Error	1564	0.0257	0.000016	Prob > F
C. Total	1569	1.1624		**<.0001***

Effect Tests

Source	Nparm	DF	Sum of Squares	F Ratio	Prob > F
Sample	2	2	1.1116	33781.64	<.0001*
1/radius	1	1	0.0004	23.7781	<.0001*
1/radius * sample	2	2	0.0007	22.7762	<.0001*

Table S3. Regression analyses of lipid and protein oxidation rate (y) on the inverse of a droplet radius (x) in sample I. (Protein: y = -8.518e-5 + 0.0076553 x, Lipid: y = 0.0320427 – 0.0014595 x)

**Analysis of Variance (Protein)**				
Source	DF	Sum of Squares	Mean Square	F Ratio
Model	1	0.00039342	0.000393	18.8235
Error	482	0.01007408	0.000021	Prob > F
C. Total	483	0.01046751		**<.0001***
**Parameter Estimates**				
Term	Estimate	Std Error	t Ratio	Prob > |t|
Intercept	-8.515e-5	0.000864	-0.10	0.9215
1/r	0.0076553	0.001764	4.34	<.0001*

**Analysis of Variance (Lipid)**				
Source	DF	Sum of Squares	Mean Square	F Ratio
Model	1	0.00001430	0.000014	0.9789
Error	482	0.00704103	0.000015	Prob > F
C. Total	483	0.00705533		0.3230
**Parameter Estimates**				
Term	Estimate	Std Error	t Ratio	Prob > |t|
Intercept	0.0320427	0.000722	44.36	<.0001*
1/r	-0.001459	0.001475	-0.99	0.3230

Table S4. Regression analyses of lipid and protein oxidation rate (y) on the inverse of a droplet radius (x) in sample II. (Protein: 0.0177008 + 0.0123076 x, Lipid: y = 0.000994 + 0.0129 x)

**Analysis of Variance (Protein)**				
Source	DF	Sum of Squares	Mean Square	F Ratio
Model	1	0.00108603	0.001086	29.8862
Error	443	0.01609806	0.000036	Prob > F
C. Total	444	0.01718408		<.0001*
**Parameter Estimates**				
Term	Estimate	Std Error	t Ratio	Prob > |t|
Intercept	0.0177008	0.001167	15.16	<.0001*
1/r	0.0123076	0.002251	5.47	<.0001*

**Analysis of Variance (Lipid)**				
Source	DF	Sum of Squares	Mean Square	F Ratio
Model	1	0.00119308	0.001193	70.2130
Error	443	0.00752761	0.000017	Prob > F
C. Total	442	0.00872070		<.0001*
**Parameter Estimates**				
Term	Estimate	Std Error	t Ratio	Prob > |t|
Intercept	0.000994	0.000798	1.25	0.2112
1/r	0.0129	0.00154	8.38	<.0001*

Table S5. Regression analyses of lipid and protein oxidation rate (y) on the inverse of a droplet radius (x) in sample III. (Protein: y = 0.0380583 + 0.0424505 x, Lipid: y = 0.0699354 + 0.0027474 x)

**Analysis of Variance (Protein)**				
Source	DF	Sum of Squares	Mean Square	F Ratio
Model	1	0.00796848	0.007968	147.9469
Error	639	0.03441678	0.000054	Prob > F
C. Total	640	0.04238526		<.0001*
**Parameter Estimates**				
Term	Estimate	Std Error	t Ratio	Prob > |t|
Intercept	0.0380583	0.001823	20.88	<.0001*
1/r	0.0424505	0.00349	12.16	<.0001*

**Analysis of Variance (Lipid)**				
Source	DF	Sum of Squares	Mean Square	F Ratio
Model	1	0.00003338	0.000033	1.9109
Error	639	0.01116119	0.000017	Prob > F
C. Total	640	0.01119457		0.1673
**Parameter Estimates**				
Term	Estimate	Std Error	t Ratio	Prob > |t|
Intercept	0.0699354	0.001038	67.37	<.0001*
1/r	0.0027474	0.001987	1.38	0.1673
